# The digital divide: amplifying health inequalities for people with severe mental illness in the time of COVID-19

**DOI:** 10.1192/bjp.2021.56

**Published:** 2021-10

**Authors:** Panagiotis Spanakis, Emily Peckham, Alice Mathers, David Shiers, Simon Gilbody

**Affiliations:** 1 Department of Health Sciences, University of York, UK; 2 Good Things Foundation, Sheffield, UK; 3 Psychosis Research Unit, Greater Manchester Mental Health NHS Foundation Trust, UK

**Keywords:** COVID-19, digital divide, digital exclusion, health inequalities, severe mental illness

## Abstract

During COVID-19, health provision and information resources have been increasingly provided via digital means (e.g. websites, apps) and this will become a standard practice beyond the pandemic. People with severe mental illness face profound health inequalities (e.g. a >20-year mortality gap). Digital exclusion puts this population at risk of heightened or compounded inequalities. This has been referred to as the ‘digital divide’. For any new digital means introduced in clinical practice to augment healthcare service provision, issues of accessibility, acceptability and usability should be addressed by researchers and developers early in the design phase, and prior to full implementation, to prevent digital exclusion.

Owing to restrictions imposed on social contact and mobility during the COVID-19 pandemic, the use of digital means as vehicles for individuals to receive health and social care, connect with and receive support from others, and spend leisure time has been accelerated at an unprecedented scale and speed. For example, many mental health services have shifted from face-to-face to remote delivery of healthcare and reports in the media suggest that mental health apps have been downloaded over 1 million times in the UK since the beginning of the pandemic. Registrations to use the National Health Service (NHS) app (a smartphone application portal for completing actions such as booking appointments and accessing medical records) increased by 111% from February to March 2020. Finally, leisure and creativity activities (e.g. museum visits, arts performances, physical activity classes and choir singing) have become accessible primarily via the internet.

A recent digital index population survey in the UK^1^ revealed the extent to which digital devices and the internet have become integral parts of many people's lives during the pandemic restrictions: 78% of participants reported that the pandemic had increased the need for digital skills in general, 54% found video-chatting and social media use to be the key digital skills for the lockdown, 51% said that digital skills had become more necessary for their home and work life and 37% reported using more technology than before to support their mental health and well-being.

Taken together, this demonstrates the enormous potential for people to benefit from the use of digital means in the time of COVID-19. More importantly, it reveals the breadth of services, activities and resources that are not easily accessible to people who are unable to use digital technology, excluding them from important resources to support their physical and mental health needs during the pandemic.

## The risk of digital exclusion among people with severe mental illness

Groups of people differ in their ability to engage with the digital world and this inequality is referred to as the digital divide. People affected by the digital divide may miss out on many of the described benefits, an experience referred to as digital exclusion. Amid the unpresented scale of digitalisation (using digital means to provide services), the risk of exclusion becomes even greater. In the UK, 7% (3.6 million people) are non-users of the internet.^1^ However, limited users of the internet may also be affected by digital exclusion.

The most common factors contributing to digital exclusion are lack of skills, lack of access/means and lack of motivation.^1^ These factors may work synergistically. For example, lack of access to the internet might hinder people from practising and improving their skills. Finding the internet too complicated might reduce motivation to engage.

Unfortunately, digital exclusion among people with severe mental illness (SMI) (schizophrenia, psychotic disorders, bipolar disorder and depression with psychotic features) has received little attention, despite this group experiencing some of the most profound health inequalities and having a life expectancy 20–25 years shorter than that of the general population.^2^ Their higher prevalence of chronic physical illnesses (such as diabetes) may further accentuate health inequalities, as the provision of self-care for such conditions becomes increasingly reliant on digital technologies. Moreover, as people with SMI are more likely to experience chronic physical illnesses, they may also need to self-isolate more often to protect themselves from COVID-19. Consequently, they may disproportionately rely on the internet and deficits in digital engagement might translate into deficits in accessing essential health and social care services. Even before the pandemic restrictions, people with SMI were at increased risk of experiencing loneliness. In the present climate of disrupted social interactions, those who are digitally excluded and are therefore unable to socialise (e.g. via social media and video calls), access information or get advice and guidance online might face an even greater risk of loneliness.

People with SMI face the same common barriers to digital inclusion as the general population, as well as additional ones linked to their mental illness.^3^ Cognitive deficits and symptoms such as hallucinations may hinder use of digital devices or the process of learning how to use them. Long periods of in-patient admission can also create gaps and a loss of touch with recent technological developments. Furthermore, the design of available digital tools (e.g. apps or websites) may not consider possible cognitive deficits or health literacy levels, limiting their usability by people with SMI, especially those with coexisting intellectual disability. Thus, accessibility is important when considering how to overcome digital inclusion barriers for people with SMI.

According to an earlier study involving people with psychosis, the digital divide in the UK is narrowing.^4^ Although this is encouraging, a sizable proportion of participants (13.8%) were digitally excluded and rates of daily internet use (56%) lagged behind rates in the general population (78%). However, more recent findings regarding people receiving community mental health rehabilitation (and hence more profoundly affected by their SMI) demonstrated that computers and the internet were used by just 17.5% and 14.4% respectively.^5^ These findings highlight digital exclusion among those more profoundly affected by their SMI. Notably, both these studies were based on a specific area of the UK prior to the COVID-19 pandemic. Thus, there is a need for broader and updated data that will capture the current situation in a nationwide representative sample of people with SMI from both primary care and secondary care mental health services covering several diagnoses and comorbidities.

Most importantly, it is difficult to determine how the COVID-19 pandemic might have affected digital exclusion in the SMI population. Speculations lead to different and often contradictory scenarios. For some non-digitally engaged people with SMI, the widespread digitalisation might have created a necessity to go online (e.g. ordering food or staying in touch with friends and family) that boosted their motivation to learn how to use the internet. For others, it might have been an overwhelming experience that reduced their motivation to engage. The financial difficulties brought by the pandemic restrictions and the closure of public spaces offering internet access (e.g. public libraries) might have exacerbated difficulties in accessing and using the internet (e.g. lack of affordability of home internet connection or digital device ownership). Finally, any negative impact of the pandemic on mental health might have intensified the digital engagement barriers that are related to the specific mental health symptoms associated with SMI.

## Reflections

Although digital exclusion among people with SMI needed to be addressed before the pandemic, the situation is now even more urgent. The advent of COVID-19 is likely to amplify inequalities, and this will persist after COVID passes. Many of the digital solutions generated by the current restrictions might become normal practice even after the pandemic has ended. This sets the scene for non-digitally engaged people with SMI to face yet another inequality for years to come, exacerbating the health and social inequalities they already experience. Research is needed to address the pressing issues in two areas. First, what is the extent of the digital divide in people with SMI and how might people be differentially affected according to key sociodemographic and health factors? And second, what are the barriers and facilitators for people with SMI in using digital technologies to support their mental and physical health needs (e.g. accessing online health information and resources), which of the common general barriers (e.g. lack of skills, access or motivation) are more prevalent in the SMI population, and what are the unique barriers that stem specifically from their complex health needs?

In the rapidly changing field of digital healthcare services, understanding the digital inclusion needs of vulnerable groups will accelerate policy and intervention changes. For example, lack of digital skills may be tackled by training programmes tailored to the specific needs of people with SMI. Lack of access would require schemes such as donation of digital devices or mobile data, and increased availability of free Wi-Fi. Government bodies and technology companies could drive these efforts forward by providing funding and organising or supporting relevant campaigns. More often than not, these solutions would need to work synergistically to address more than one barrier at the same time. We suggest that healthcare services used by people with SMI should focus on both facilitating digital engagement (e.g. members of the care team that the person is familiar with could provide support) and continuing to offer non-digital alternatives for those not yet able to engage with digital services.

As a final point, we would urge all stakeholders involved in forums where digital services are designed and developed to consider issues of accessibility and usability of these services for people with SMI. This should be done at the outset and be an integral part of the design process.

Life and work will continue to be digitalised, with many anticipated benefits for those who are able to engage with the digital world. For others, this raises the risk of yet another form of health and social inequality. Supporting people with SMI to be digitally included is a matter of tackling inequality and improving quality of life and as such should be integral in our work with our patients and research participants.

## Data Availability

Data availability is not applicable to this article as no new data were created or analysed in this study.

## References

[1] Lloyds Bank. Lloyds Bank UK Consumer Digital Index 2020. Lloyds Bank, 2020.

[2] Hayes HF, Marston L, Walters K, King BM, Osborn JPD. Mortality gap for people with bipolar disorder and schizophrenia: UK-based cohort study 2000–2014. Br J Psychiatry 2017; 211: 175–81. 28684403 10.1192/bjp.bp.117.202606 PMC5579328

[3] Greer B, Robotham D, Simblett S, Curtis H, Griffiths H, Wykes T. Digital exclusion among mental health service users: qualitative investigation. J Med Internet Res 2019; 21: e11696. 30626564 10.2196/11696 PMC6329420

[4] Robotham D, Satkunanathan S, Doughty L, Wykes T. Do we still have a digital divide in mental health? A five-year survey follow up. J Med Internet Res 2016; 18: e309. 27876684 10.2196/jmir.6511 PMC5141335

[5] Tobitt S, Percival R. Switched on or switched off? A survey of mobile, computer and internet use in a community mental health rehabilitation sample. J Ment Health 2019; 28: 1, 4–10. 28675329 10.1080/09638237.2017.1340623

